# In situ FRET-based localization of the N terminus of myosin binding protein-C in heart muscle cells

**DOI:** 10.1073/pnas.2222005120

**Published:** 2023-03-13

**Authors:** Jessica Chandler, Conor Treacy, Simon Ameer-Beg, Elisabeth Ehler, Malcolm Irving, Thomas Kampourakis

**Affiliations:** ^a^Randall Centre for Cell and Molecular Biophysics, and British Heart Foundation Centre of Research Excellence, King’s College London, London SE1 1UL, United Kingdom; ^b^Richard Dimbleby Laboratory of Cancer Research, School of Cancer & Pharmaceutical Sciences, King’s College London, London SE1 1UL, United Kingdom; ^c^School of Cardiovascular and Metabolic Medicine and Sciences, British Heart Foundation Centre of Research Excellence King's College London, London SE1 1UL, United Kingdom

**Keywords:** cardiac myosin, myosin binding protein-C, phosphorylation, foerster resonance energy transfer, TCSPC-FLIM

## Abstract

Cardiac myosin binding protein-C (cMyBP-C) is a thick filament–associated regulatory protein frequently found mutated in patients suffering from hypertrophic cardiomyopathy (HCM). Recent in vitro experiments have highlighted the functional significance of its N-terminal region (NcMyBP-C) for heart muscle contraction, reporting regulatory interactions with both thick and thin filaments. To better understand the interactions of cMyBP-C in its native sarcomere environment, in situ Foerster resonance energy transfer–fluorescence lifetime imaging (FRET–FLIM) assays were developed to determine the spatial relationship between the NcMyBP-C and the thick and thin filaments in isolated neonatal rat cardiomyocytes (NRCs). In vitro studies showed that ligation of genetically encoded fluorophores to NcMyBP-C had no or little effect on its binding to thick and thin filament proteins. Using this assay, FRET between mTFP conjugated to NcMyBP-C and Phalloidin-iFluor 514 labeling the actin filaments in NRCs was detected by time-domain FLIM. The measured FRET efficiencies were intermediate between those observed when the donor was attached to the cardiac myosin regulatory light chain in the thick filaments and troponin T in the thin filaments. These results are consistent with the coexistence of multiple conformations of cMyBP-C, some with their N-terminal domains binding to the thin filament and others binding to the thick filament, supporting the hypothesis that the dynamic interchange between these conformations mediates interfilament signaling in the regulation of contractility. Moreover, stimulation of NRCs with β-adrenergic agonists reduces FRET between NcMyBP-C and actin-bound Phalloidin, suggesting that cMyBP-C phosphorylation reduces its interaction with the thin filament.

## Significance

Cardiac myosin binding protein-C (cMyBP-C) is one of a group of sarcomeric proteins that have been frequently implicated in the development of heart disease and heart failure. However, the molecular mechanisms underlying contractile regulation by cMyBP-C are poorly understood. In this study, we used a newly developed fluorescence lifetime-based Foerster resonance energy transfer assay (FRET) approach to monitor cMyBP-C’s regulatory interactions in the native environment of the intact sarcomere lattice of isolated cardiac muscle cells. We show that multiple populations of cMyBP-C exist during the relaxed state of cardiac muscle, reflecting thin and thick filament–bound states of its N-terminal domains, which are further modulated by phosphorylation. Our findings have important implications for heart muscle function in health and disease.

The cardiac isoform of myosin binding protein-C (cMyBP-C) has emerged as an important regulator of contractility both in the healthy heart and in disease (1). Mutations in the gene encoding for cMyBP-C (MYBPC3) are the second most common cause of inheritable Hypertrophic Cardiomyopathy (HCM) in the human population, and knockout of cMyBP-C leads to the development of cardiomyopathies and heart failure in transgenic animal models. It follows that the native structure and function of cMyBP-C are essential for normal performance and energy efficiency in the heart (2).

**Fig. 1.**
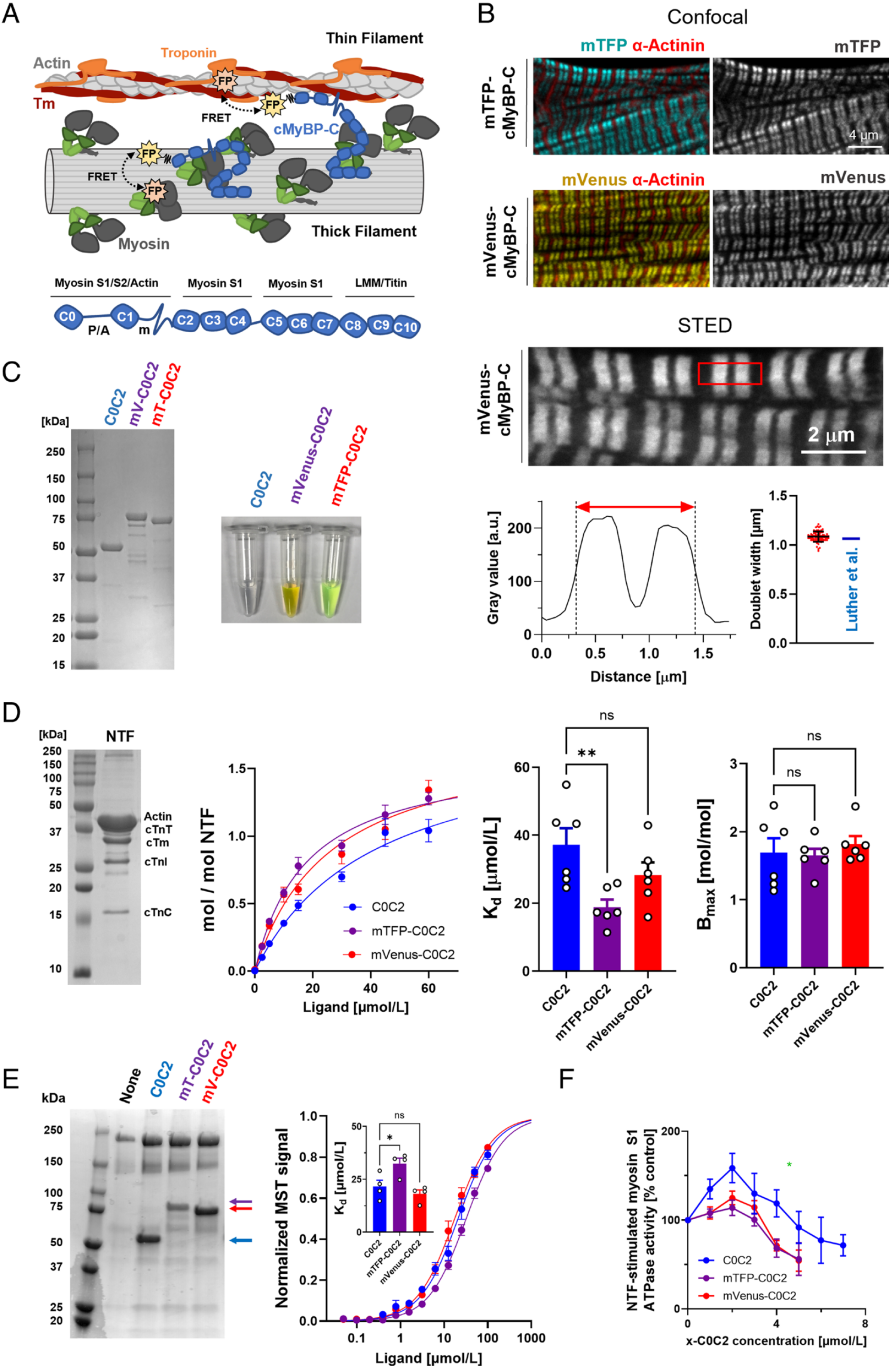
In vitro validation of cMyBP-C-based FRET biosensors. (*A*, *Top*) Schematic illustration of the cMyBP-C-based FRET assay. Genetically encoded fluorescent proteins (FP) conjugated to the N-terminus of cMyBP-C (blue) can FRET (black dashed double arrows) with FP incorporated into either thick or thin filament components depending on its localization. (*Bottom*) Domain architecture and known protein interaction partners of cMyBP-C. (*B*, *Top*) Sarcomeric localization of cMyBP-C N-terminally conjugated to either mTFP (cyan) or mVenus (yellow) expressed in neonatal rat cardiomyocytes (NRCs) determined by confocal microscopy. NRCs were counterstained against α-actinin (red) to visualize Z-discs. (*Bottom*) Super-resolution STED microscopy analysis of the sarcomeric localization of mVenus-cMyBP-C in NRC. A line scan for a single sarcomere is shown below the image with the doublet full width half maximum indicated by a red double arrow. (*C*) Sodium dodecyl-sulfate polyacrylamide gel electrophoresis (SDS-PAGE) and image of purified wild type, mTFP-, and mVenus-C0C2. (*D*) Native thin filament (NTF) cosedimentation assays with wild-type (blue), mTFP- (purple), and mVenus-C0C2 (red). (*E*, *Left*) Myosin cosedimentation of C0C2, mTFP-C0C2, and mVenus-C0C2. (*Right*) Normalized Microscale Thermophoresis (MST) curves for C0C2, mTFP-C0C2, and mVenus-C0C2 binding to myosin S2Δ. (*F*) Dose–response curve for the effect of C0C2, mTFP-C0C2, and mVenus-C0C2 on the NTF-stimulated cardiac myosin S1 ATPase activity. Means ± SEM, n = 3–6. Statistical significance of differences between groups was assessed with one-way ANOVA followed by Tukey’s post hoc test: *P < 0.05, **P < 0.01, and ns – not significant.

cMyBP-C is composed of eight Ig-like and three fibronectin-like domains termed C0 through C10 (Fig. 1*A*). Some of these domains are connected by isoform-specific linkers, including the proline/alanine-rich (P/A) region between domains C0 and C1, and the phosphorylatable m-motif connecting domains C1 and C2. The C-terminal domains of cMyBP-C anchor it to the myosin-containing thick filaments, where it is localized to nine stripes in the central region of each half filament called the “C zone”. The 43-nm periodicity of these stripes matches the helical periodicity of the myosin motors and is likely determined by strong interactions with the myosin tails and with the periodic super-repeats of the scaffold protein titin in the filament backbone (3, 4). The central domains of cMyBP-C bind to the myosin motors (5, 6), stabilizing the OFF state in which they are folded back against their tail domains in the thick filament backbone in an asymmetric configuration called the interacting-heads motif (IHM), inhibiting the interaction of the motors with actin in the thin filaments that drives contraction (7). The IHM has also been associated with a “super-relaxed” state of myosin, in which the intrinsic ATPase rate of the myosin motors is greatly reduced (8).

The N-terminal domains of cMyBP-C (NcMyBP-C) can bind not only to myosin motor and tail domains but also to the thin filaments (9). The interactions with the thick filaments are believed to stabilize the OFF state and reduce contractility, whereas thin filament binding has generally been associated with an activating effect (10, 11). Thin filaments are activated during each heartbeat by calcium binding to troponin, which displaces tropomyosin from its resting position in which it blocks myosin binding to actin. NcMyBP-C binding stabilizes the activated state of tropomyosin, increasing the calcium sensitivity of the thin filaments. The interactions of NcMyBP-C with multiple thick and thin filament components are enabled by the flexibility conferred by the largely unstructured P/A linker and m-motif (12, 13).

These interactions are likely to depend on the regulatory states of the thin and thick filaments but are also controlled by phosphorylation of the m-motif at three or more sites by multiple protein kinases including protein kinase A (PKA), protein kinase C, and protein kinase D (14, 15). Phosphorylation of cMyBP-C has been associated with a decrease in myofilament calcium sensitivity and an increase in the rates of force development and mechanical relaxation (16, 17), effects that may be mediated by decreased binding of NcMyBP-C to the thin and thick filaments. Ablation of the PKA phosphorylation sites in cMyBP-C in transgenic mouse models leads to heart disease and heart failure (18).

The gene encoding for cMyBP-C (MYBPC3) is frequently mutated in patients suffering from HCM. Some of these are missense mutations in which the altered protein is incorporated into the myofilaments (19) with altered binding affinity of its N-terminal domains to thin or thick filaments (20, 21), suggesting that these interactions are essential for normal function. Point mutations that increase the actin affinity of NcMyBP-C prolong contraction and delay relaxation in animal models, hallmarks of HCM (20).

Although the studies described above provide very strong evidence that the interactions of NcMyBP-C with thin and thick filament proteins and their modulation by cMyBP-C phosphorylation are essential for the normal function of the heart and can be impaired in heart disease, the multiplicity of these interactions and their regulation (15) has impeded understanding of the underlying mechanisms. In vitro studies with isolated proteins are generally confined to a single binding partner and do not preserve the native environment of the sarcomere lattice in which multiple interactions can occur in dynamic regulatory states. However, recent spectroscopic studies of the intra- and intermolecular interactions of isolated cMyBP-C with both actin and myosin have elucidated some the potential regulatory mechanisms involved (22, 23). Transgenic animal models do preserve the native structural and functional environment of the sarcomere but generally focus on the functional effects of single point mutations and do not allow experimental control of the many other parameters that modulate cMyBP-C’s interactions with thin and thick filaments.

Given that context, we sought to develop an approach with the potential to answer the most fundamental question about the interaction of cMyBP-C with thin and thick filaments in the native sarcomere of a heart muscle cell: Are its N-terminal domains bound to thin filaments or to thick filaments? To do so, we expressed a modified cMyBP-C with a genetically encoded fluorescent protein conjugated to its N terminus in neonatal rat cardiomyocytes (NRCs) and used Foerster resonance energy transfer (FRET) to estimate its proximity to a second fluorophore bound to different sites on the thin and thick filaments in the same cells. We show that there is a population of NcMyBP-C molecules close to the thin filaments in relaxed heart muscle cells, corresponding to the diastolic state in the beating heart, but other NcMyBP-Cs are close to the thick filaments. Both strong myosin head binding to actin and β-adrenergic stimulation leading to cMyBP-C phosphorylation reduce the fraction of NcMyBP-C molecules close to the thin filaments. Our results lead to a molecular model of the interactions of cMyBP-C with thin and thick filaments in the native sarcomere lattice and of the effects of cMyBP-C phosphorylation, providing a framework for a molecular understanding of the dynamic regulatory function of cMyBP-C in the working heart and its implications for heart disease.

## Results

### Design and In Vitro Validation of cMyBP-C-based FRET Biosensors.

To monitor the proximity of NcMyBP-C to either the thin or thick filaments in intact cardiomyocytes by FRET, we conjugated its N terminus to the genetically encodable fluorescent proteins mTFP or mVenus (Fig. 1*A*). A complete list of all developed FRET biosensors and their sarcomeric localizations is shown in Table 1.

**Table 1.** Summary of average FRET efficiencies <E%> for all donor–acceptor pairs

			Relaxed			Rigor		
Donor (Localization)	Acceptor (Localization)	ISO	Mean	SEM	N	Mean	SEM	N
mTFP-cMyBP-C	mVenus-cRLC (*myosin heads and thick filament*)	−	2.35	0.84	19	0.29	0.49	19
	mVenus-cTnT (*troponin and thin filament*)	−	−1.24	0.68	17	1.56	0.79	18
	Phalloidin-iFluor 514 (*F-actin and thin filament*)	−	12.00	0.63	39	9.87	0.65	31
	Phalloidin-iFluor 514 (*F-actin and thin filament*)	+	8.55	0.66	24	8.39	0.40	32
mTFP-cTnT (troponin, thin filament)	mVenus-cMyBP-C	−	6.37	0.90	14	2.39	0.93	19
	Phalloidin-iFluor 514 (*F-actin and thin filament*)	−	19.54	0.69	24	14.51	0.65	29
mTFP-cRLC (myosin heads, thick filament)	mVenus-cRLC (*myosin heads and thick filament*)	−	3.86	0.44	20	2.26	0.64	21
	mVenus-cTnT (*troponin and thin filament*)	−	2.11	0.66	19	1.39	0.50	20
	mVenus-cMyBP-C	−	1.38	0.60	20	2.11	0.75	20
	Phalloidin-iFluor 514 (*F-actin and thin filament*)	−	9.33	0.47	26	7.36	0.43	29
mTFP-(AA)_15_-mVenus	−	16.24	0.81	29	18.55	0.64	29	

Means ± SEM, n = 9–39.

Both fluorescent cMyBP-C constructs localized to a pair of stripes in each myosin-containing A-band of the sarcomere when expressed in intact neonatal rat cardiomyocytes (NRCs) by transient transfection (Fig. 1*B*). We used stimulated emission depletion (STED) super-resolution microscopy (*Lower*) to define the location of the labeled cMyBP-C more precisely, showing that each doublet band had a full width half maximum (FWHM) of 1.09 ± 0.05 μm (mean ± SD, n = 75). This is very close to that expected from the localization of cMyBP-C determined by immunoelectron microscopy of isolated rat cardiac muscle cells, with a doublet FWHM of ~1.06 μm (3).

We tested for possible functional effects of conjugation of mTFP or mVenus to the N terminus of cMyBP-C using bacterially expressed N-terminal fragments of cMyBP-C containing domains C0 through C2 (C0C2) conjugated to one of the fluorescent proteins at its N terminus (Fig. 1*C*). Cosedimentation assays showed that fluorophore incorporation had only small effects on C0C2 binding to isolated native thin filaments (NTFs) (Fig. 1*D*) (24). mTFP-C0C2 did have a higher affinity for NTFs than C0C2 alone, with K_d_ values of 18 ± 2 μmol/L and 42 ± 8 μmol/L (means ± SEM, n = 6), respectively, but the binding stoichiometry as indicated by B_max_ was unchanged. Similar results were obtained in assays for binding of the C0C2 constructs to myosin. The conjugates readily cosedimented with artificial filaments of bovine cardiac myosin (Fig. 1*E*). We quantified the binding affinity of the C0C2 constructs for a fragment of the S2 region of myosin consisting of its first 128 residues (myosin S2Δ) using a Microscale Thermophoresis assay (25). The K_d_ for mVenus-C0C2 was similar to that of native C0C2 (K_d_ of ~ 20 μmol/L, *Inset*), whereas that for mTFP-C0C2 was slightly lower (K_d_ of ~ 35 μmol/L).

We also investigated the effects of fluorophore conjugation to C0C2 on the actomyosin interaction by measuring the stimulation of cardiac myosin S1 ATPase activity of native thin filaments (NTFs; Fig. 1*F*). Unmodified C0C2 shows a biphasic dose–response relationship in this assay, with an increase in ATPase of about 60% up to 2 μmol/L C0C2, followed by a decrease at higher concentrations with an EC_50_ of ~4 μmol/L, as shown previously (26). mTFP and mVenus conjugation blunted the activating effect, but the inhibitory effect had an EC_50_ similar to that of native C0C2.

Taken together, these results suggest that conjugation of mTFP and mVenus to the N terminus of cMyBP-C has minimal effects on the cellular localization and function of cMyBP-C and that these biosensors are suitable for the proposed FRET measurements.

### In Situ FRET Screen of cMyBP-C Donor with Thin and Thick Filament–based Acceptors.

**Fig. 2.**
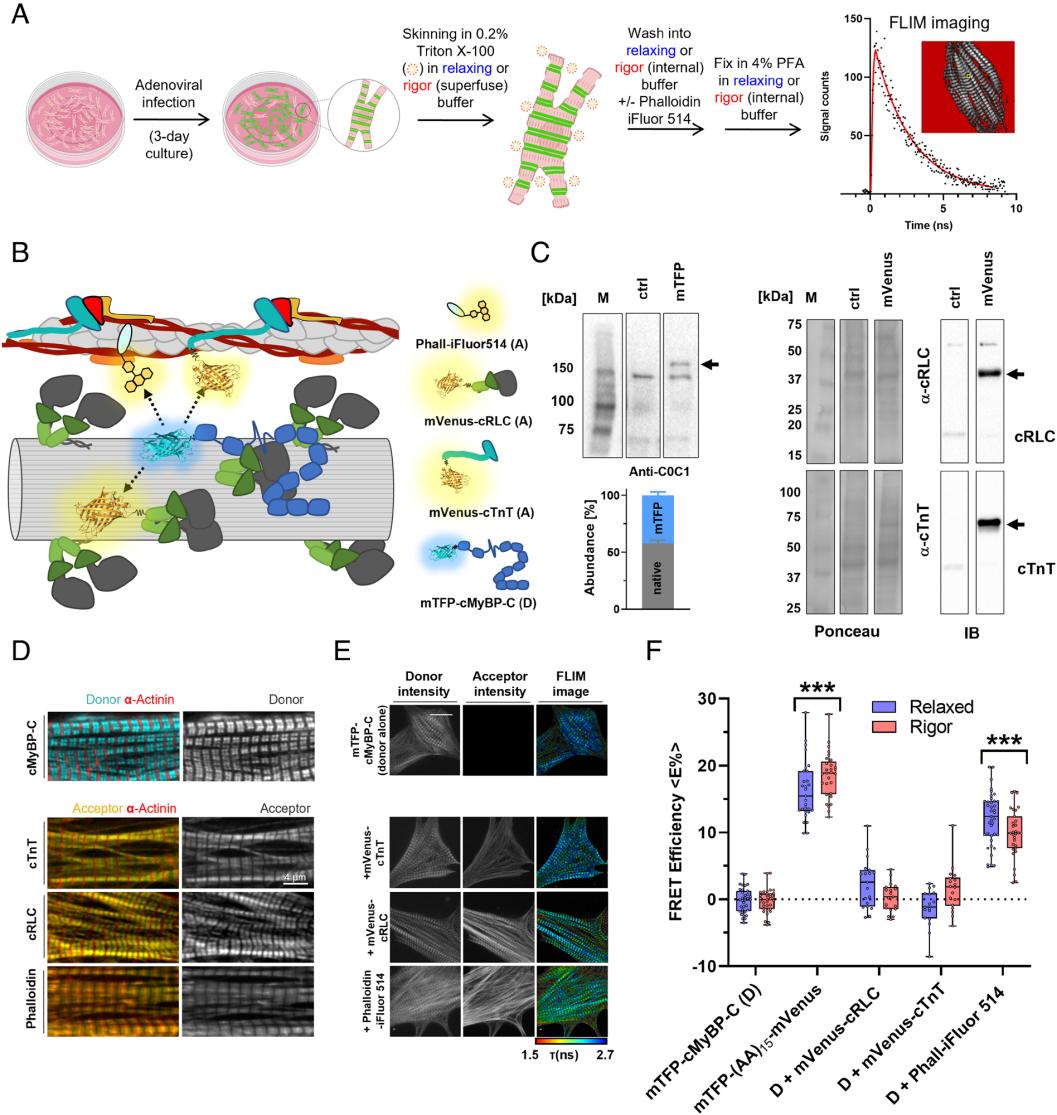
In situ FRET screen of mTFP-cMyBP-C with mVenus-conjugated sarcomeric protein acceptors or iFluor514-labeled Phalloidin. (*A*) Workflow for FRET–FLIM assays in demembranated cardiomyocytes. (*B*) Cartoon representation of the FRET screening assay. mTFP-cMyBP-C (FRET donor, D) was coexpressed with mVenus-cRLC, mVenus-cTnT, or Phalloidin-iFluor514 (FRET acceptors, A) was added to demembranated NRCs as FRET acceptors. (*C,* *Left*) Expression level of endogenous and mTFP-conjugated cMyBP-C in NRCs after adenoviral infection estimated by Western blot against NcMyBP-C. (*Right*) Expression level of endogenous and mVenus-conjugated cRLC and cTnT in NRCs. (*D*) Localization of mTFP-cMyBP-C, mVenus acceptor proteins, and Phalloidin-iFluor514 in NRCs determined by confocal microscopy. (*E*) Representative fluorescence lifetime images (FLIM) of mTFP-cMyBP-C alone or coexpressed with mVenus acceptor proteins or Phalloidin-iFluor514. (*F*) Summary of FRET efficiencies for all tested mTFP-cMyBP-C and acceptor pairs in the relaxed (blue) or rigor state (red) of NRCs. Means ± SEM, n = 9–31 cells from n = 3–6 independent NRC preparations. Statistical significance of differences between groups was assessed with a two-way ANOVA followed by Sidak’s post hoc test: ***P < 0.001.

Next, we tested three different mVenus FRET acceptors, looking for changes in mTFP-cMyBP-C donor fluorescence lifetime that would indicate FRET (Fig. 2*A*). We coexpressed mTFP-cMyBP-C donor in NRCs with either mVenus-conjugated myosin regulatory light chain (cRLC) in the thick filaments or mVenus-cTnT acceptor in the thin filaments via adenoviral infection. In addition, we used Phalloidin-iFluor514 to label thin filament actin in NRCs expressing mTFP-cMyBP-C alone (Fig. 2 *A* and *B*).

We tested for any functional effects of mVenus incorporation into myosin regulatory light chain by exchanging recombinantly produced mVenus-cRLC into isolated cardiac myofibrils (CMFs), replacing about 80% of the endogenous cRLC (*SI Appendix*, Fig. S1). The ATPase activity of cRLC-exchanged CMFs at both low (pCa 9) and high (pCa 4.5) Ca^2+^ concentrations was identical to that of native CMFs, suggesting that mVenus incorporation into cRLC had no effect on myosin and actomyosin ATPase activity, or on its Ca^2+^ regulation. Additionally, we stoichiometrically replaced the endogenous cRLC of isolated cardiac myosin with mVenus-cRLC (*SI Appendix*, Fig. S2) and performed single nucleotide turnover experiments using synthetic myosin filaments formed from either native or cRLC-exchanged myosin. Both the amplitudes and time constants of the fast and slow phases of ATP release from synthetic myosin filaments corresponding to the disordered (DRX) and super-relaxed (SRX) states of myosin, respectively, were unaffected by mVenus incorporation. Moreover, similar to the binding experiments with native myosin described above (Fig. 1*E*), mTFP-C0C2 readily cosediments with mVenus-cRLC-exchanged cardiac myosin (*SI Appendix*, Fig. S3), suggesting that fluorophore incorporation into both cMyBP-C and myosin had little effect on their interactions.

Adenoviral infection of NRCs resulted in replacement of about 40% of the endogenous cMyBP-C with the mTFP-conjugated protein (Fig. 2 *C*, *Left*), whereas mVenus-cRLC and mVenus-cTnT constructs stoichiometrically replaced the endogenous proteins (Fig. 2 *C*, *Right*). All acceptor constructs correctly localized in intact NRCs as shown by confocal microscopy (Fig. 2*D*), and we confirmed precise colocalization of mVenus-cTnT and mVenus-cRLC to the thin and thick filaments, respectively, by STED super-resolution microscopy (*SI Appendix*, Fig. S4 *A* and *B*). Following adenovirus-mediated expression of the fluorescent constructs, NRCs were demembranated by mild detergent treatment in either relaxing solution (pCa 9) containing 25 μmol/L nitro-blebbistatin or rigor solution, and were fixed with paraformaldehyde for subsequent fluorescence lifetime imaging microscopy (FLIM) (Fig. 2*A*). Average sarcomere lengths were ~1.85 μm and ~1.7 μm in relaxing and rigor conditions, respectively (*SI Appendix*, Fig. S4*C*). FLIM images generally had a single cardiomyocyte in the field of view (Fig. 2*E*), but typically 6 to 10 cells were imaged per dish to assess inter-cell variability. Donor fluorescence lifetime distributions were well described by a single exponential function (*SI Appendix*, Fig. S5). Fluorescence lifetime histograms for each image were used to calculate the intensity-weighted mean fluorescence lifetime of the donor in the presence of acceptors (τ_DA_) and converted into FRET efficiencies (<E%>) using the average fluorescence lifetime of the donor alone from the same set of experiments (τ_D_) as described in the *Materials and Methods* *section*.

As a positive control, we expressed mTFP conjugated to mVenus via a 15–amino acid flexible linker in NRCs using adenoviral infection. The maximum separation of the mTFP and mVenus fluorophores in a structural model of this construct is ~10 nm, which is larger than the Foerster radius (R_0_) of ~6 nm for this FRET pair assuming random orientation of the excitation and emission dipoles (κ^2^ = 2/3). The measured average intensity-weighted FRET efficiency was ~16–18% (Fig. 2*F* and Table 1), corresponding to a mean donor–acceptor separation of ~8 nm, in reasonable agreement with the structural model.

Coexpression of mVenus-cRLC or mVenus-cTnT as acceptor with mTFP-cMyBP-C as donor did not significantly reduce its fluorescence lifetime in relaxing or rigor conditions (Fig. 2*F* and Table 1), i.e., no FRET was detected. This does not necessarily imply that the donor–acceptor separation was too large (> 15 nm) for FRET; it is also possible that the relative stoichiometry of these acceptors was too low. We therefore introduced an alternative acceptor that is likely to have high relative stoichiometry by labeling actin with Phalloidin-iFluor514 in NRCs containing mTFP-cMyBP-C donor. This reduced the donor lifetime corresponding to <E%> values of ~12% and ~10% in the relaxed and rigor states, respectively (Fig. 2*F* and Table 1). These results show that some NcMyBP-Cs are close enough to the actin filaments for energy transfer in relaxing conditions and that the number of such molecules is likely reduced when myosin binds strongly to actin in rigor. To exclude the possibility that Phalloidin labeling changed the interaction between cMyBP-C and thin filaments, we performed cosedimentation experiments of mTFP-C0C2 with Phalloidin-iFluor514-labeled native thin filaments. The results show that labeling thin filaments with the small-molecule probe had no effect on its interaction with NcMyBP-C (*SI Appendix*, Fig. S6).

We also took the alternative approach of using mTFP conjugated to either cRLC or cTnT as the FRET donor. Like the mVenus-conjugated proteins, mTFP-cRLC and mTFP-cTnT correctly localized to thick and thin filaments, respectively (*SI Appendix*, Fig. S7). Coexpression of mTFP-cRLC with mVenus-cRLC produced a small but reproducible reduction in donor fluorescence lifetime with an estimated FRET efficiency of ~4% in relaxing conditions, which was reduced to ~2% in rigor (*SI Appendix*, Fig. S8), suggesting that the N termini of the two cRLCs in each myosin are on average slightly further apart when the motor domains bind to actin in rigor than in the relaxed state. The fluorescence lifetime of mTFP-cTnT was significantly reduced by coexpression with mVenus-cMyBP-C corresponding to a FRET efficiency of ~6% in relaxing conditions.

This shows that although some NcMyBP-Cs are close to the thin filament in relaxing conditions, on average, they do not bind near the N terminus of cTnT, as expected from the different spacings of the two molecules along the filaments.

### In Situ Calibration of FRET Signals with Phalloidin-iFluor514 as Acceptor.

**Fig. 3.**
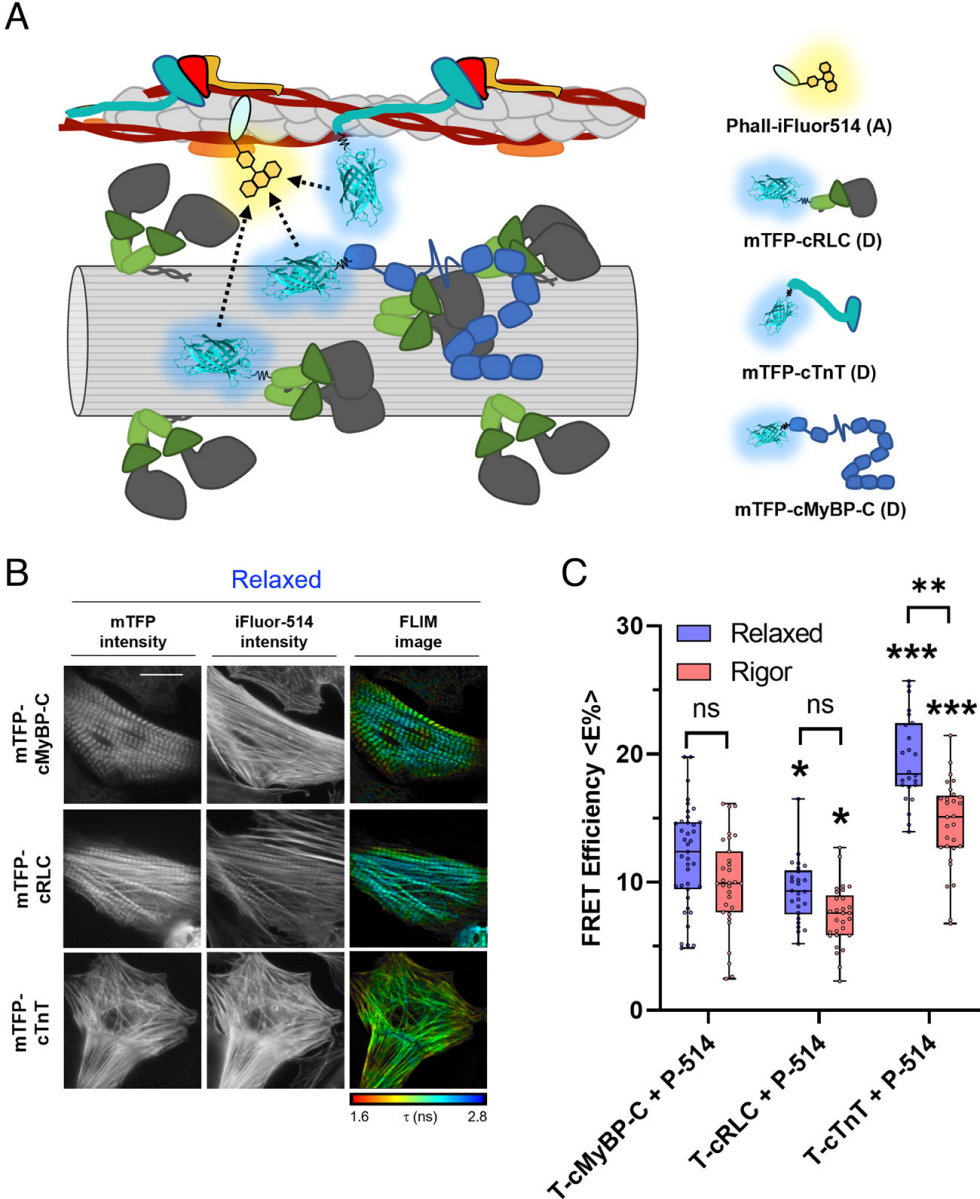
FLIM experiments with Phalloidin-iFluor514 as the acceptor. (*A*) Cartoon representation of the acceptor FLIM assay. NRCs expressing mTFP conjugated to cRLC, cTnT, or cMyBP-C (FRET donors, D) were stained with Phalloidin-iFluor514 as the FRET acceptor (*A*). (*B*) Representative FLIM images of mTFP-cMyBP-C, mTFP-cRLC, and mTFP-cTnT in the presence of Phalloidin-iFluor514. (*C*) Average FRET efficiencies for mTFP-cMyBP-C, mTFP-cRLC, and mTFP-cTnT in the presence of Phalloidin-iFluor514 as the acceptor in the relaxed (blue) or rigor state (red) of NRCs. Means ± SEM, n = 29–31 cells from n = 4–6 preparations. Statistical significance of differences vs. mTFP-cMyBP-C, or relaxed vs. rigor conditions were assessed with a two-way ANOVA followed by Tukey’s post hoc test: *P < 0.05, **P < 0.01, and ***P < 0.001.

Quantitative interpretation of FRET efficiencies between protein components in the sarcomeric filament lattice is complicated by the multiple copies of each protein within and between filaments, so that any given donor–acceptor pair will be separated by a multiplicity of different distances in the 3D lattice corresponding to the multiple copies of the labeled proteins, and each separation will correspond to a different FRET efficiency. When comparing different donor–acceptor pairs, this complexity is compounded by possible differences in donor–acceptor stoichiometry. Although the observation of significant FRET between a NcMyBP-C donor and Phalloidin acceptor shows that there is a population of NcMyBP-C molecules close to the thin filaments in relaxing conditions, it is difficult to estimate the size of that population or the distance distribution from the FRET efficiency. As a step toward overcoming this problem, we therefore made an internal comparison of the FRET efficiencies measured for mTFP-NcMyBP-C with those for mTFP-cRLC and mTFP-cTnT on the surface of the thick and thin filaments, respectively, with Phalloidin-iFluor514 bound to F-actin as the acceptor in all cases (Fig. 3*A*). This approach allows a calibration of the results for NcMyBP-C with respect to those from well-defined thick and thin filament components, with the same spatial distribution of actin acceptor sites in the sarcomere in all cases. Representative FLIM images for the three different FRET pairs are shown in Fig. 3*B*.

We found that there was significant FRET between mTFP-cRLC in the thick filaments and Phalloidin-iFluor514 on actin (Fig. 3*C* and Table 1); <E%> was about 10% in relaxing conditions. <E%> for the cRLC donor was significantly less than that for cMyBP-C donor in both relaxing and rigor conditions, but not zero, indicating that the interfilament gap is sufficiently small, and the mTFP probe sufficiently large, for some mTFP-cRLCs to come sufficiently close to some Phalloidin–actins for FRET, even in relaxing conditions. The higher FRET efficiency observed with the NcMyBP-C donor then suggests that on average, the N terminus of cMyBP-C is slightly closer to actin than that of cRLC or a greater fraction of NcMyBP-Cs are close to actin. As expected, <E%> for mTFP-cTnT in the presence of the Phalloidin acceptor is significantly larger than that for NcMyBP-C (<E%> of about 20%), suggesting that on average, the N-terminal extension of cTnT is closer to actin. Moreover, <E%> for the cTnT–Phalloidin pair is decreased when myosin motors bind strongly to actin in rigor, suggesting that the N-terminal extension of cTnT moves away from the thin filament surface, likely mediated by the movement of tropomyosin on the surface of the thin filament.

### Effect of β-Adrenergic Stimulation on the Proximity of cMyBP-C to Actin In Situ.

**Fig. 4.**
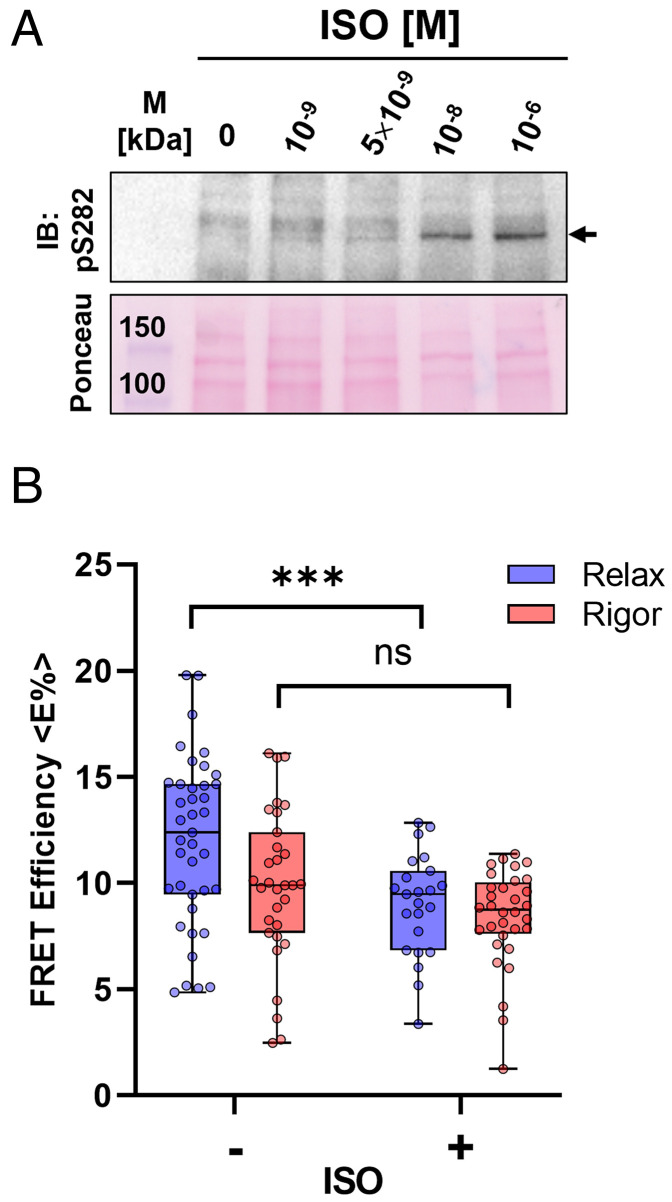
Effect of isoproterenol (ISO) treatment on FRET efficiency of the mTFP-cMyBP-C–Phalloidin-iFluor514 FRET pair. (*A*) ISO dose–response curve for cMyBP-C phospANOVAorylation on serine 282 in intact NRCs. (*B*) Average FRET efficiencies for the mTFP-cMyBP-C–Phalloidin-iFluor514 pair in untreated (−) and ISO-treated (+) NRCs in relaxed (blue) or rigor conditions (red). Means ± SEM, n = 24–31 cells from n = 4–6 preparations. Statistical significance of differences between untreated and ISO-treated NRCs was assessed with a two-way ANOVA followed by Sidak’s post hoc test: ***P < 0.001 and ns – not significant.

Phosphorylation of cMyBP-C by PKA downstream of β-adrenergic receptor stimulation is an important regulator of cardiac muscle performance, and heart failure is frequently associated with cMyBP-C hypophosphorylation (27). We therefore investigated the effects of β-adrenergic stimulation on the measured FRET efficiency between NcMyBP-C and Phalloidin-labeled actin by incubating intact NRCs with isoproterenol (ISO) before the skinning and fixation steps. ISO treatment of NRCs increased cMyBP-C phosphorylation in a dose-dependent manner, with maximal phosphorylation at ~1 μmol/ L [ISO] (Fig. 4*A*). ISO treatment of NRCs led to a reduction in <E%> between mTFP-cMyBP-C and Phalloidin-iFluor514 in relaxing conditions (Fig. 4*B* and Table 1), suggesting that phosphorylation reduces the fraction of NcMyBP-Cs close to actin but had no effect on the <E%> measured in rigor conditions.

## Discussion

### Use of FRET Probes as Structural Biosensors in the Intact Sarcomere.

Ligation of genetically encoded fluorescent proteins to sarcomeric protein components for FRET measurements provides a potentially powerful approach to investigate the interactions and dynamics of the labeled proteins in the intact functioning sarcomere. For this approach to provide accurate information about the native sarcomere, it is clearly important that the presence of the fluorescent protein does not alter the native structure or function of the proteins being investigated. Here, we show that conjugation of mTFP or mVenus to the N terminus of cMyBP-C had no effects on its sarcomeric localization and binding stoichiometries to other sarcomeric proteins in vitro, although it slightly changed the binding affinity in some cases (Fig. 1). Since the first ten N-terminal residues of cMyBP-C are largely disordered in solution (28), the linker to the conjugated fluorescent proteins is likely to be relatively flexible.

In the case of cTnT, although the N terminus of this protein is important for thin filament activation and sarcomere function through its binding to the tropomyosin overlap region (29–31), conjugation of the fluorescent protein GFP to the N terminus of cTnT has been shown to have no or little effect on cardiac myofilament function in both isolated cardiac myofibrils and intact cardiomyocytes (32). Similarly, GFP conjugation to the N terminus of cRLC was shown to have no effect on heart muscle contractility either in vivo or in vivo (33, 34). Non-muscle myosin II carrying a RLC N-terminally fused to GFP is also normally regulated in the presence of F-actin, suggesting that the GFP did not interfere with formation of the myosin OFF state (35). Consistent with that conclusion, mVenus-RLC incorporation into either CMF or isolated cardiac myosin had no effect on myosin/actomyosin ATPase activity and its Ca^2+^ regulation, formation of the super-relaxed state of myosin, and the interaction of myosin with mTFP-conjugated cMyBP-C (*SI Appendix*, Figs. S1, S2 and S3). The N-terminal extensions of both cRLC and cTnT are intrinsically disordered and likely to be highly dynamic and mobile (36–38). Taken together, these results suggest that these N-terminal intrinsically disordered regions of sarcomeric proteins are suitable attachment points for genetically encoded fluorescent proteins in spectroscopic studies on isolated cardiomyocytes. These unstructured or intrinsically disordered regions are likely able to accommodate the large fluorescent proteins without significantly altering their protein–protein interactions, dynamics, or associated functions.

Likewise, our use of Phalloidin-iFluor514 as a thin filament acceptor probe depends on it not perturbing the native structure or function of the thin filaments. Recent cryo-EM reconstructions showed that this is the case; Phalloidin binding to the cleft between individual actin protomers had no effect on the overall filament structure (39, 40). Addition of saturating concentrations of Phalloidin-iFluor514 had no effect on mTFP-C0C2 binding to isolated thin filaments (*SI Appendix*, Fig. S6), suggesting that this small molecule is a suitable probe of intermolecular FRET experiments in the intact sarcomere lattice.

The FRET efficiencies measured in the present experiments depend strongly on the relative orientation of the donor and acceptor fluorescence dipoles, and this dependence is generally expressed in terms of the orientation factor κ^2^. A common assumption is that the dipoles are randomly orientated, giving a κ^2^ value of 2/3 (41). However, the organization of the thin and thick filaments in the sarcomere is highly anisotropic, so this assumption might not be valid in the current study. In fact, polarized fluorescence measurements showed that rhodamine probes conjugated to Phalloidin are orientationally ordered with respect to the filament axis in isolated cardiac myofibrils (42), suggesting that the iFluor514 acceptor probe in our experiments might be similarly anisotropic.

In contrast, the fluorescent proteins conjugated to the N-termini of cRLC, cTnT, and cMyBP-C are likely to be isotropic as a result of the disordered and flexible linkers discussed above. Moreover, unconjugated mTFP expressed in NRCs has a largely diffuse localization with some unspecific Z-disc staining (*SI Appendix*, Fig. S7), demonstrating that mTFP does not bind directly to either the thin or thick filaments, which might have resulted in a preferred orientation. Finally, fluorescence polarization experiments with bifunctional rhodamine probes attached to the N-terminal extension of cRLC in demembranated rat ventricular trabeculae showed an isotropic orientation of the probe under all conditions tested (36). It is therefore unlikely that the FRET results presented here were influenced by changes in the orientation factor.

Probably the most important limitation of interpretation of FRET measurements in the sarcomere is the multiplicity of labeled proteins along and between filaments in the sarcomeric lattice together with the limited information about the stoichiometry of labeling. Multiple FRET acceptors close to a donor can increase the effective Foerster radius R_0_, for example, via the “antenna effect” of “FRET surplus” (41), based on statistical selection of more favorable relative dipole orientations among the acceptor molecules. More generally, the multiplicity of labeled proteins and unknown stoichiometry preclude the use of FRET efficiencies to determine distance distributions for pairs of sarcomeric proteins. The effect of variable donor labeling stoichiometry was largely eliminated in the present experiments by measuring donor lifetime in the presence of an acceptor, Phalloidin-iFluor514, with high labeling stoichiometry and very high local concentration compared to that of the donors. This allowed an in situ calibration of the FRET efficiency <E%> for the different donor-labeled proteins of interest.

FRET binding assays of this general type could be used in future studies to determine the effects of heart disease–associated mutations, sarcomere-directed small-molecule effectors, and physiological interventions on the structure and function of cardiac sarcomeric proteins.

### Distribution of the N Terminus of cMyBP-C between Thin and Thick Filaments in Relaxed Cardiac Muscle Cells.

Previous biochemical experiments have shown that isolated N-terminal domains of cMyBP-C can bind to both thin and thick filament components with low micromolar affinity in vitro (15, 43–45). Because of the high local or effective concentrations of potential binding partners in the sarcomere lattice, these in vitro studies predict that native NcMyBP-C is more likely to be bound to either thin or thick filaments rather than free in solution. However, in vitro studies with isolated proteins cannot account for the steric constraints imposed by the myofilament lattice, and conformational disorder of cMyBP-C in situ might lead to a fraction of NcMyBP-Cs being within the interfilament space (46). Such a location would, however, not exclude the possibility of that fraction being bound to myosin head domains.

The current results present direct evidence for binding of NcMyBP-C to the thin filaments by demonstrating energy transfer between a fluorescent donor attached to the N terminus of cMyBP-C and an acceptor on actin (Fig. 2). This conclusion was supported by the observation of energy transfer between a donor on cTnT and an acceptor on the N terminus of cMyBP-C (*SI Appendix*, Fig. S8). Although it is not possible to estimate the fractions of NcMyBP-C bound to thin and thick filament sites from the FRET data, the observation that energy transfer between NcMyBP-C and Phalloidin is much less than that between cTnT and Phalloidin (Fig. 3*C*) suggests that a large fraction of NcMyBP-Cs are either bound to thick filaments or in the interfilament space.

The present results therefore shed new light on the binding of NcMyBP-C to the thick filaments. No FRET signal was detected between mTFP attached to domain C0 and mVenus attached to cRLC (Fig. 2*F*) or vice versa (*SI Appendix*, Fig. S8). This result suggests that NcMyBP-C does not bind directly to cRLC in the intact sarcomere lattice despite the fact that domain C0 does bind to the cRLC with micromolar K_d_ in vitro (28). C0 also interacts weakly with the myosin motor domain (K_d_ of ~90 μmol/L) (5), so it is possible, given the high-effective myosin concentration, that this interaction dominates in the intact lattice with full-length proteins.

**Fig. 5.**
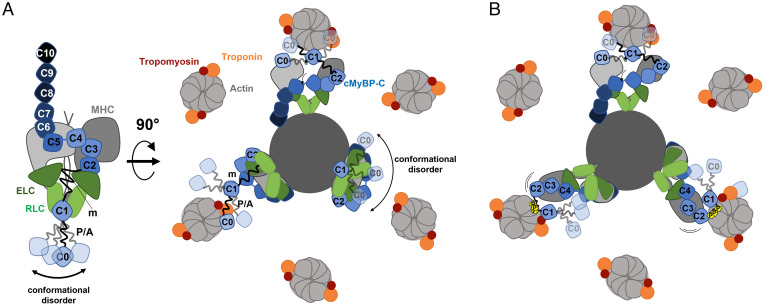
Model for the arrangement of cMyBP-C domains in the myofilament lattice. (*A*, *Left*) The interaction between cMyBP-C (blue) and cardiac myosin (gray). (*Right*) Transverse view of the model showing the arrangement of cMyBP-C domains in relation to thin and thick filaments in relaxed heart muscle. (*B*) Proposed effect of cMyBP-C phosphorylation on its interactions with thick and thin filaments. For details, see main text.

These results in combination with those of previous studies lead to the model for the conformation of cMyBP-C in relaxed cardiac muscle shown in Fig. 5*A*. The C-terminal domains (C8-C10) of cMyBP-C are tightly bound to the myosin tail domains and titin on the thick filament surface, anchoring the protein to the sarcomeric C-zone in stripes with a 43-nm periodicity (3, 4, 47). The central domains (C2C7) bind tightly to the myosin head domains in vitro (5), suggesting that domains C2 through C10 are localized to the thick filament in its OFF state (Fig. 5*A*).

In contrast, domains C0 and C1 have binding sites for both thin and thick filament components and are likely mobile and highly dynamic, a feature which may be related to the largely unstructured P/A linker and m-motif, leading to conformational disorder of the N terminus of cMyBP-C in both the bound and unbound states (12, 13). According to the present results, some NcMyBP-Cs would be bound to the thin filament via interactions of domains C0 and C1, whereas a large fraction remain close to the thick filament or occupy positions within the interfilament space (Fig. 5*A*). This model is consistent with previous results showing that at least a fraction of cMyBP-Cs are bound to the thin filament in relaxing conditions (46, 48) and that others are bound to other sarcomere components or free in solution.

### Effects of Myosin Head Binding to Actin and cMyBP-C Phosphorylation.

Although <E%> for the cRLC–Phalloidin pair slightly decreased from the relaxed to the rigor state by about 2%, the difference was not statistically significantly (*P* = 0.24 for a two-way ANOVA followed by Tukey’s post-hoc test). One possible mechanism that might produce a difference in this direction would be that mTFP conjugated to the N-terminal extension of the RLC is more mobile in the relaxed state of the myofilaments and can partially occupy positions in the interfilament space. In contrast, its position is more restricted during rigor when the majority of myosin heads are bound to actin and block the interfilament space (49). Alternatively, a change in orientation of the myosin lever arm from the relaxed to rigor conformation might contribute to the small changes in FRET efficiency.

These results therefore suggest that the radial separation between cRLC and the thin filament is similar in the relaxed or OFF state and rigor state in which myosin motors are strongly bound to actin. This is consistent with our previous fluorescence polarization studies which showed that the cRLC has a similar conformation in the rigor and relaxed states and with the hypothesis that the cRLC might act as a thick filament-docking domain for myosin motors that catalyzes the formation of the OFF state at the end of the working stroke (50, 51).

In contrast, <E%> for the cTnT–Phalloidin pair decreased in the transition to rigor, consistent with some N-terminal regions of cTnT (NcTnT) moving away from the thin filament surface when myosin motors bind to actin in rigor. Myosin motors might directly compete with the NcTnT for binding sites on actin, or the azimuthal movement of tropomyosin might release this region of cTnT from the tropomyosin overlap region (37). These results are consistent with a model in which the NcTnT is in a conformational equilibrium between thin filament–bound and thin filament–unbound states in relaxing conditions, with the former probably stabilizing the thin filament OFF state. Myosin motor binding to actin may shift the conformational equilibrium of NcTnT toward the unbound state, further destabilizing the OFF state of the thin filament by cooperative interactions along its length (37).

Similarly, strong myosin motor attachment to actin in rigor decreased <E%> for the cMyBP-C–Phalloidin pair, suggesting that, on average, the N terminus of cMyBP-C moves away from the thin filament surface in rigor. However, some NcMyBP-C molecules may remain bound to the thin filaments in rigor as indicated by a <E%> of about 10%, in agreement with electron microscopy studies using immunolabeling of cMyBP-C in rigor myofibrils (52). Domains C0 and C1 of MyBP-C have been shown by cryo-EM reconstructions to bind to isolated thin filaments in positions that would block myosin motor binding to actin (53), suggesting that the reduced <E%> might be due to direct competition between myosin heads and NcMyBP-C for binding sites on actin. Further work will be required to investigate the physiological relevance of this observation. The great majority of myosin motors are bound to thin filaments in rigor (49), whereas probably only about 10% are bound at the peak of the cardiac twitch in isolated rat trabeculae (54, 55).

Phosphorylation of cMyBP-C has important functional consequences for the myocardium, including increased myosin motor kinetics and relaxation, enhanced length-dependent activation, and changes in myofilament calcium sensitivity (17, 56). Here, we show that the ISO treatment–mediated phosphorylation of the m-motif of cMyBP-C in intact myofilaments reduces the fraction of NcMyBP-Cs in proximity to the thin filaments in the relaxed state (Fig. 4*B*), in agreement with in vitro experiments showing reduced binding of the phosphorylated N-terminal domains of cMyBP-C to isolated native thin filaments (24).

This result has important implications for the mechanism of myofilament regulation by cMyBP-C phosphorylation because previously proposed mechanisms, which were mainly based on in vitro experiments using isolated proteins, suggested that the main role of phosphorylation was to *increase* the fraction of NcMyBP-Cs bound to thin filaments (15). However, the present results can be reconciliated with the in vitro data in a model in which phosphorylation increases the fraction of myosin motors and NcMyBP-C bound to the thin filaments coupled to a structural rearrangement of its N-terminal domains that leaves domain C0 further away from the thin filament surface, likely occupying positions in the interfilament space or binding to the myosin heads (Fig. 5*B*). This model is also consistent with the results of FRET and electron microscopy studies on the structural effects of phosphorylation on the N-terminal domains of cMyBP-C (13, 57). Phosphorylation has been proposed to result in a disorder-to-order transition in the m-motif that leaves the N-terminal domains in a more closed conformation. Phosphorylation of cMyBP-C might therefore regulate the mode of interaction between its N-terminal domains and actin. In the unphosphorylated state, both the P/A linker and m-motif are in extended and flexible conformations, allowing the N-terminal domains of cMyBP-C to bridge the interfilament gap and dynamically interact with actin without perturbing the OFF state of the myosin motors. In contrast, the PKA phosphorylation–induced structural rearrangement of the m-motif would lead to an interaction with the thin filament that brings motors closer to their actin binding sites. The consequence would be a destabilization of the thick filament OFF state and increased mobility of the motors, facilitating their interaction with the thin filaments and thereby enhancing contractile function.

## Materials and Methods

### Protein Production.

Proteins were expressed in BL21(DE3)-RIPL cells conjugated to an N-terminal histidine tag and TEV protease cleavage site and purified as described previously (15).

### Cosedimentation Experiments and Microscale Thermophoresis.

Preparation of bovine native thin filaments (NTFs) and cosedimentation and Microscale Thermophoresis experiments were performed as described previously (15). Cardiac bovine myosin was prepared from the bovine left ventricle, and cosedimentation experiments performed as described in ref. 26.

### Single Nucleotide Turnover Experiments.

Purified bovine β-cardiac myosin was exchanged with mVenus-cRLC as described previously (58). Native and cRLC-exchanged cardiac myosin were diluted to 0.4 μmol/L in reaction buffer (10 mmol/L PIPES, pH 7, 5 mmol/L MgCl_2_, and 1 mmol/L DTT) and incubated on ice for 2 h to form synthetic myosin filaments. 50 μL myosin filament solution per well was aliquoted into a black 384-well plate (GREINER). Mant-ATP was added to a final concentration of 0.8 μmol/L using automated injector units; the system was allowed to age for 90 s, and 2 mmol/L Adenosine-5′-triphosphate (ATP) was added using the automated injectors. Fluorescence intensity was continuously sampled every 1 s for 15 min. Data were fitted to a biexponential decay function extracting the fraction of the slow (SRX state) and fast (DRX state) phases.

### Native Thin Filament–Myosin S1 and Myofibrillar ATPase Activity Measurements.

Bovine cardiac myosin S1 (Cytoskeleton Inc.), NTFs, and C0C2 proteins were dialyzed and/or gel-filtered into assay buffer (composition in mmol/L: 15 PIPES, 5 MgCl_2_, and 1 DTT) containing 100 µmol/L CaCl_2_ (pCa 4). Final concentrations of NTFs and myosin S1 for ATPase assays were fixed at 10 µmol/L and 0.02 µmol/L, respectively. C0C2 was added in the concentration range between 0 and 8 µmol/L, and the mixture was equilibrated at 25 °C for 5 min. The reaction was started by addition of ATP to a final concentration of 1 mmol/L in 100 µL final assay volume. ATPase activity was monitored on a real-time basis using ATPase ELIPA Biochem Kit (Cytoskeleton Inc.) according to manufacturer’s instructions. Assays were conducted in a 96-well format by reading the absorbance at 360 nm every 30 s for a duration of 30 min using a ClarioStar plate reader system (BMG Labtech).

Native cRLC was extracted from myofibrils reconstituted with mVenus-RLC and ATPase activity at low (pCa 9) and high (pCa 4.5) free Ca^2+^ concentration measured as described previously (59).

The cardiac isoforms of rat, human, and bovine myosin, actin, troponin, and cMyBP-C share about 90% sequence identity and more than 95% sequence similarity.

### Cell Culture, Transient Transfection, and Adenoviral Infection.

Neonatal rat cardiomyocytes (NRCs) were prepared and cultured as described previously (60). Transient transfections were carried out using Escort III (SIGMA) as the transfection agent. Adenoviral vectors for the expression of mTFP- or mVenus-conjugated proteins were sourced from VectorBuilder. Adenoviral infection was performed by direct addition of the > 10^10^ PFU/mL adenovirus stock supplied by VectorBuilder (1:500–50,000) to the NRC media, and cells incubated for overnight or 1 h, respectively, at 37 °C and 5 % CO_2_. Media were then replaced with culture medium, and NRCs incubated for 0 to 2 d.

NRCs were skinned following a protocol adapted from ref. 61. Cell culture media were removed, and the cells were washed into relaxing (superfuse) or rigor (superfuse) solution at 37 °C for 10 min. The cell membrane was then permeabilized by the addition of 0.2 % (v/v) Triton X-100 directly to the superfuse solution for 1.5 min at room temperature. After skinning, cardiomyocytes were washed into relaxing (internal) solution or rigor (internal) solution. The buffers were made up in 10 mmol/L HEPES with 120 mmol/L K-propionate added to adjust the ionic strength. The relaxing buffer was prepared to include an ATP-regenerating system containing 10 mM phosphocreatine and 5 mmol/L ATP, a calcium concentration close to the value observed during diastole in intact cardiomyocytes (50 nmol/L free Ca^2+^, pCa 7.3) to limit thin filament activation and 25 μmol/L (S)-nitro-blebbistatin. For cells in relaxing conditions, NRCs were incubated in culture media supplemented with 25 μmol/L (S)-nitro-blebbistatin for 1 h prior to skinning. The rigor buffer was prepared lacking ATP to prevent the detachment of myosin heads from actin and also lacking calcium and containing 10 mmol/L etheylenediaminetetraacetic acid (EDTA) to prevent overcontraction and chelate Mg^2+^ to drive rigor formation. Additionally, 10 mmol/L L-glutathione was added in the buffers to protect the skinned cells against oxidative stress, and 4% (w/v) dextran (MW 40,000) was included to osmotically compress the myofilament lattice of the skinning cardiomyocytes to physiological lattice spacing.

### Western blotting.

Wet blot transfer at 60 mA (20 to 30 V) was performed overnight to blot the proteins from gels run by SDS‐PAGE onto nitrocellulose membranes in 1 × SDS‐PAGE transfer buffer using Mini-PROTEAN Tetra Cell and blotting module components. Correct transfer was confirmed by ponceau red staining for 10 min at room temperature. Nonspecific binding sites were then blocked by incubation of the membrane in 5% (w/v) nonfat dry milk or 3% (w/v) BSA in SDS‐PAGE low-salt buffer for 1 h at room temperature. Primary (1°) antibodies (rabbit anti-GFP (Abcam ab6556, 1:5,000), mouse anti-pS282 cMyBP-C (ENZO, ALX-215-057-R050; 1:1000), rabbit anti-MLC2 (Abcam, ab92721), and rabbit antitroponin T (Santa Cruz Biotechnology, sc-20025, 1:5,000)), secondary (2°) horseradish peroxidase (HRP)–conjugated antibodies (HRP goat anti-rabbit IgG (Calbiochem, AP187P, 1:2,000), and HRP rabbit anti-mouse IgG (Dako, P0260, 1:5,000)) and Precision Protein StrepTactin-HRP Conjugate, to visualize the marker, were diluted at the appropriate concentration in 5% (w/v) nonfat dry milk or 3% (w/v) bovine serum albumin (BSA) in low salt (LS) buffer, and each incubated at room temperature for 1 h or at 4 °C overnight. Three times 5-min washing steps were performed after each incubation in 5% (w/v) nonfat dry milk or 3% (w/v) BSA in LS buffer and LS buffer alone, respectively. Finally, membranes were developed in a chemiluminescence reaction on the enzyme HRP conjugated to the 2° antibodies by incubating the membranes in Clarity Western ECL Substrate (BIORAD) for 4 min, according to the manufacturer’s instructions. Results were visualized on the ChemiDoc MP Imaging System using ImageLab software. Fiji software was used for densitometric analysis of western blot bands.

### Confocal and STED Microscopy.

Immunostaining was performed with the following primary and secondary antibodies: mouse anti-α-actinin (Sigma, A7811, 1:500 dilution), rabbit anti-C0C1 (kindly provided by Mathias Gautel; 1:1,000 dilution), Cy3 AffiniPure goat anti-mouse IgG (Jackson, 115-165-146, 1:500 dilution), Cy5 AffiniPure goat anti-mouse IgG (Jackson, 115-175-146, 1:500 dilution), and DyLight-549 goat anti-rabbit (Vector Laboratories, STAR36D54, 1:100 dilution). All confocal imaging was performed at the Nikon Imaging Centre, King’s College London, using a Nikon A1R confocal mounted on a Nikon Ti-E Eclipse microscope with Nikon's Perfect Focus System and equipped with a 60×/1.40 NA oil immersion lens. NIS-Elements software was used for acquisition in an optical configuration comprising sequential scanning of the 488.6-nm, 562.0-nm, and 639.6-nm laser lines and DU4 detection unit (four standard photomultiplier tubes) at 1,024 × 1,024 resolution, 1.62 × zoom, and 8 × image averaging.

STED imaging was performed on the Abberior STEDYCON coupled to a Leica SP5 II confocal microscope equipped with a 100×/1.44 NA oil immersion lens. Abberior STEDYCON smart control software was used for imaging with the STED 775-nm laser line, set at 42-nm resolution, and a fixed pixel size of 25 nm. Cells were immune-stained with an anti-GFP primary antibody (Abcam, ab6556;1:100 dilution) followed by a ATTO647N goat anti-rabbit IgG (Sigma, 18373; 1:50 dilution).

### FLIM Data Acquisition and Analysis.

Time-domain multiphoton fluorescence lifetime imaging was performed with a multiphoton FLIM system built around a Nikon Eclipse Ti-E, which was fit with a 40× 1.30 NA Nikon Plan-Fluor oil objective. The Ti-E was equipped with spectral filter cubes obtained from Semrock™. Prior to FLIM, cells were imaged in wide-field epifluorescence illumination, 1280 × 1024 pixels and pixel size 6.37 px/μm, to confirm expression of both FRET probes. The mTFP1 donor was visualized with 448 ± 20-nm excitation filter, 442-nm dichroic mirror, and a 482 ± 20-nm emission filter, while the mVenus or iFluor 514 acceptors were visualized with 510 ± 10-nm excitation filter, 514-nm dichroic mirror, and a 542 ± 27-nm emission filter. The multiphoton FLIM time-correlated single photon counting (TCSPC) measurements were taken using a 480 ± 30-nm emission filter (Semrock) and an HPM 100-40 hybrid detector (Becker & Hickl) using a Ti:Sapphire laser pulsing at 80 MHz (Coherent Chameleon Vision II) tuned to 875 nm (two-photon excitation wavelength for mTFP1). Laser power was adjusted to give average photon counting rates of the order 10^4^ to 10^5^ photons s^–1^ (0.0001 to 0.001 photons/excitation event) and with peak rates approaching 10^5^ photons s^–1^ below the maximum counting rate afforded by the TCSPC electronics to avoid pulse pileup. The multiphoton FLIM images were acquired at 8 x zoom, 256 × 256 pixels, and fast scan speed. The TCSPC images taken on 8x scan speed have an effective pixel size of 5.02 px/μm. Acquisition times of the order of 300 s and low excitation power were used to achieve sufficient photon statistics for fitting while avoiding either pulse pileup or significant photobleaching.

All fitting of the FLIM data was performed in TRI2 analysis software. Multiphoton FLIM images were first preprocessed with thresholding 10 to 100 % to remove background and 9 × 9 pixel binning to increase signal count in donor fluorescence decay transients. The decay transients from the binned pixels were fit with the monoexponential or biexponential Levenberg–Marquardt (LM) algorithm to determine the fluorescence lifetime (Tau; τ), as previously reported for fixed and live-cell studies (62).

Multiphoton intensity and fluorescence lifetime images (.sdt) were converted into 8-bit plane images (.jpg) and images merged (no blending) to create composite FLIM images. Fluorescence lifetime histograms for each image were exported from TRI2 analysis software as .txt files, and data imported into excel for analysis. For each experiment, n = 3–8 donor alone (D) fluorescence lifetime histograms were normalized to 1 and summed. This summed fluorescence lifetime histogram was used to calculate the intensity-weighted average D fluorescence lifetime (τ_D_). For each individual image, the donor in the presence of acceptor (D+A) fluorescence lifetime histogram was used to calculate the intensity-weighted D+A fluorescence lifetime (τ_DA_). For FRET–FLIM in NRCs, n = 3–5 experiments (n = 9–30 individual images) were performed for each donor and acceptor combination.

FRET efficiencies (<E%>) were calculated for each individual D+A fluorescence lifetime (τ_DA_) against the average D fluorescence lifetime (τ_D_) from the same experiment using Eq. **1** as follows:

[1] $$<E\;\%>=(1-(\frac{t_{\mathrm{DA}}}{t_{D}}))*100.$$

## Supplementary Material

Appendix 01 (PDF)

## Data Availability

All study data are included in the article and/or *SI Appendix*.
