# Supplementary material for: In situ FRET-based localization of the N terminus of myosin binding protein-C in heart muscle cells
Source: Proc Natl Acad Sci U S A. 2023 Mar 13;120(12):e2222005120. doi: 10.1073/pnas.2222005120 (PMC10041117; doi:10.1073/pnas.2222005120)
Supplement: Supplementary file 1 — Appendix 01 (PDF) [file pnas.2222005120.sapp.pdf]

Supporting Information for

In situ FRET-based localization of the N-terminus of myosin binding protein-C in heart muscle cells

Jessica Chandler, Conor Treacy, Simon Ameer-Beg, Elisabeth Ehler, Malcolm Irving and Thomas Kampourakis*

Corresponding author: Thomas Kampourakis
Email: thomas.kampourakis@kcl.ac.uk

This PDF file includes:

Figures S1 to S8

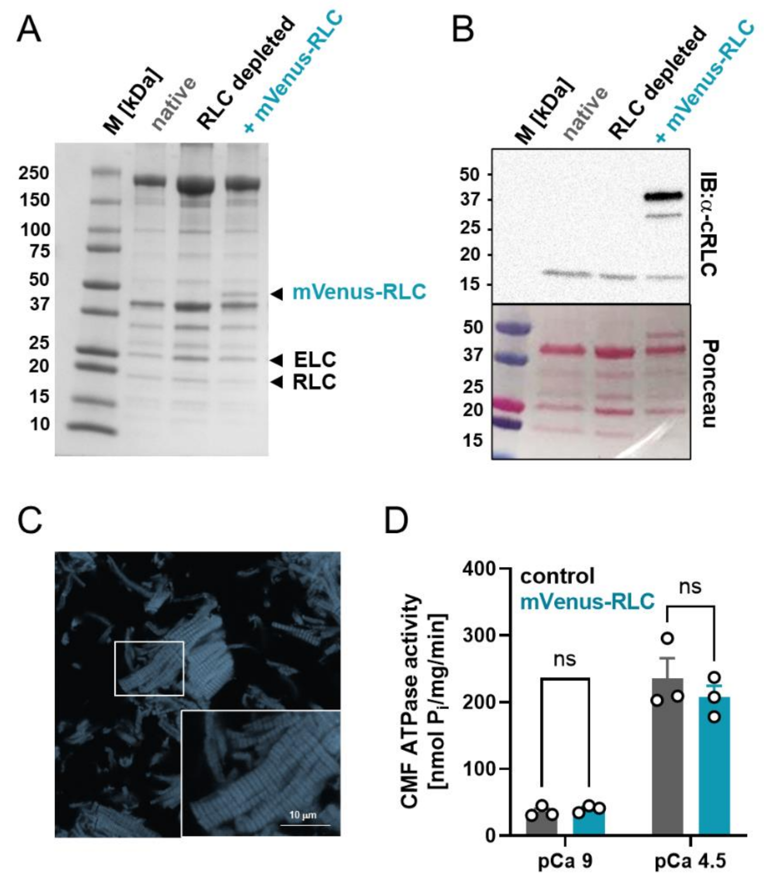

Fig. S1. Effect of mVenus-RLC incorporation on cardiac myofibrillar ATPase activity. (A) SDS-PAGE of native, RLC depleted and mVenus-RLC reconstituted bovine cardiac myofibrils (bCMF). RLC, ELC and mVenus-RLC are labelled accordingly. (B) Western-blot against RLC of samples from (A). More than 80% of the endogenous RLCs in bCMF were replaced by mVenus-RLCs. (C) Confocal image of RLC-exchanged bCMF confirming A-band localization of mVenus-RLC. (D) ATPase activity of native (grey, control) and mVenus-RLC exchanged bCMF (cyan) at low (pCa 9) and high Ca2+ concentrations (pCa 4.5). Means ± SEM, n=3. Statistical significance of differences were assessed with an unpaired, student's t-test: ns – not significant.

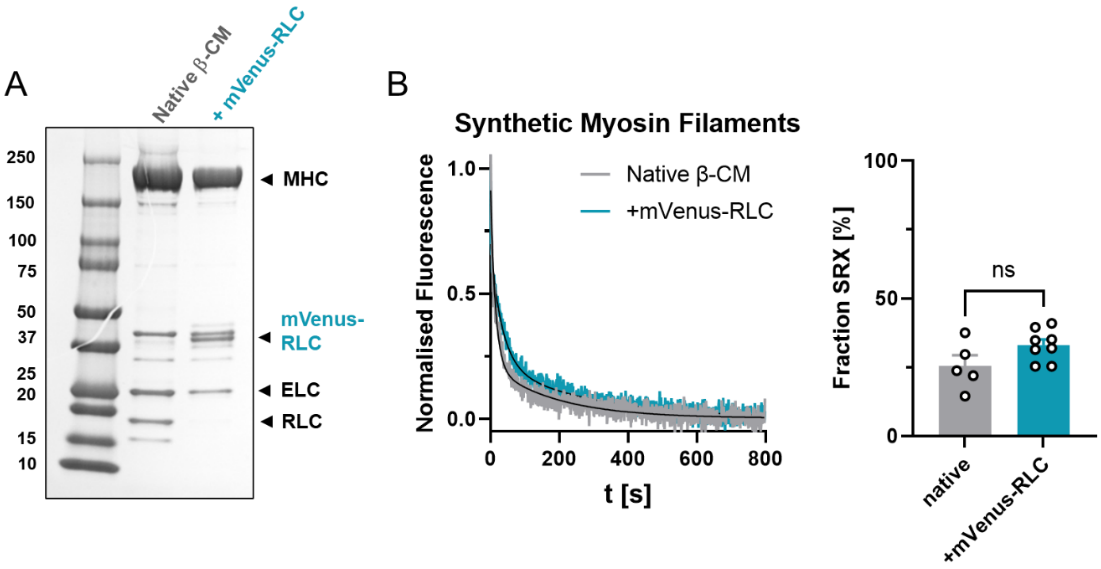

Fig. S2. Effect of mVenus-RLC incorporation on cardiac myosin super-relaxed state (SRX). (A) SDS-PAGE of native and mVenus-RLC exchanged bovine cardiac myosin. Myosin heavy chain (MHC), essential light chain (ELC), regulatory light chain (RLC) and mVenus-RLC are labelled accordingly. Please note that the exchange protocol stoichiometrically replaced the endogenous RLC with mVenus-RLC. (B) Left: Example traces of mant-ATP pulse chase experiments using synthetic myosin filaments assembled from native bovine cardiac myosin (grey) and bovine cardiac myosin exchanged with mVenus-RLC (cyan). Bi-exponential fits are shown as black continuous lines. Right: Fraction of myosin heads in the SRX state extracted from the bi-exponential fits for both native and mVenus-RLC-exchanged synthetic thick filaments. Means ± SEM, n=5-8. Statistical significance of differences was assessed with an unpaired, student's t-test: ns – not significant.

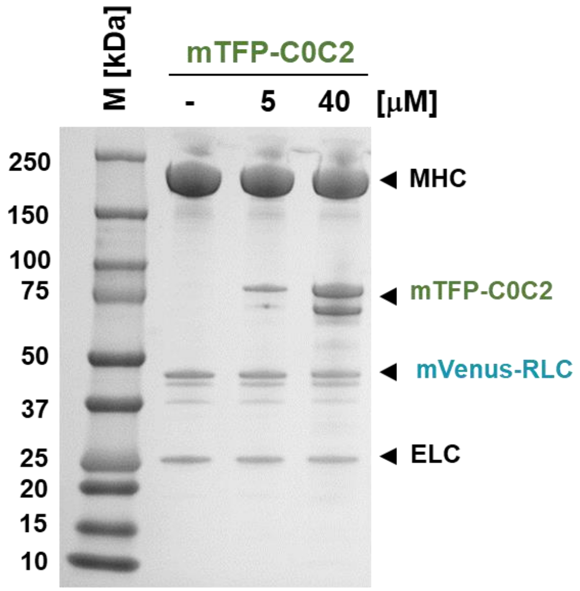

Fig. S3. Co-sedimentation of mVenus-RLC exchanged β-cardiac myosin with mTFP-C0C2.

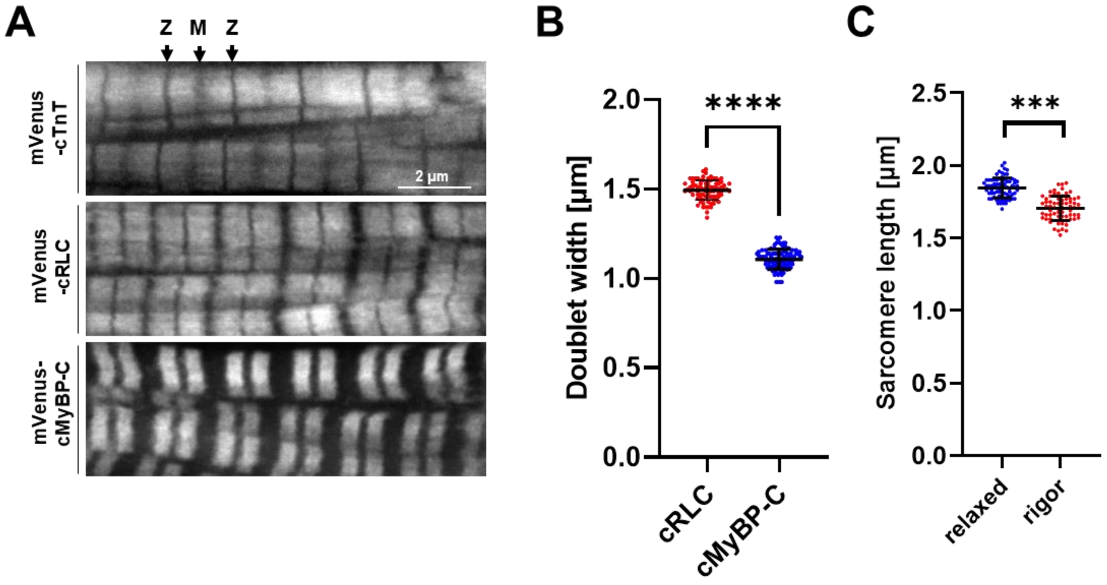

Fig. S4. (A) Representative STED images of mVenus-cTnT, mVenus-cRLC and mVenus-cMyBP-C expressed in neonatal rat cardiomyocytes. M-band and Z-disk are labelled accordingly. (B) Comparison of the doublet width (FWHM) of mVenus-cRLC and mVenus-cMyBP-C signal. (C) Sarcomere length of demembranated NRCs in relaxing (blue) and rigor conditions (red) measured by confocal microscopy.

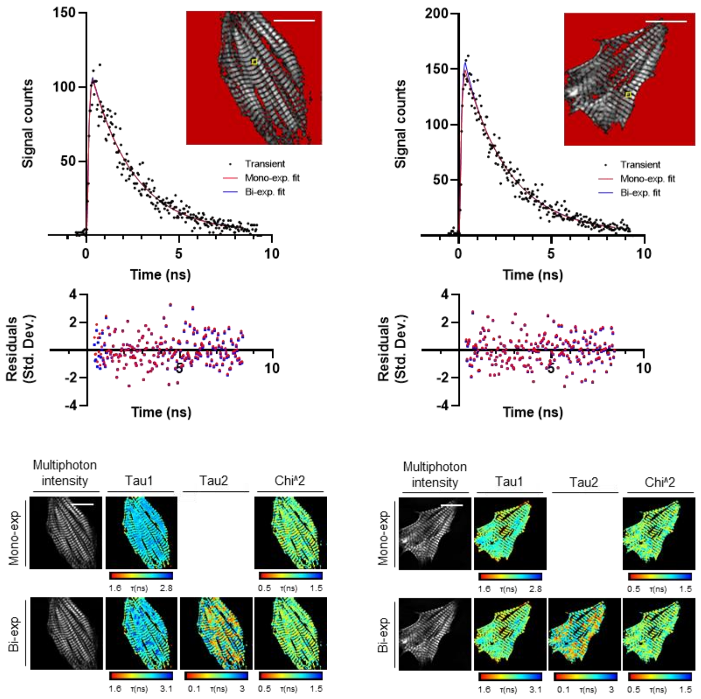

	Tau1 (ns)	Tau2 (ns)	Chi^2
Mono	2.44	n/a	1.00
Bi	2.5	2.333	0.95

	Tau1 (ns)	Tau2 (ns)	Chi^2
Mono	2.248	n/a	0.97
Bi	2.335	2.188	0.97

Comparison of Fits	
Simpler model	One phase decay
Probability it is correct	78.02%
Alternative model	Two phase decay
Probability it is correct	21.98%
Ratio of probabilities	3.549
Preferred model	One phase decay
Difference in AICc	-2.533

Fig. S5. Comparison of mono- and bi-exponential fits to mTFP-cMyBP-C fluorescence lifetime in the absence (left) and in the presence (right) of Phalloidin-iFluor514.

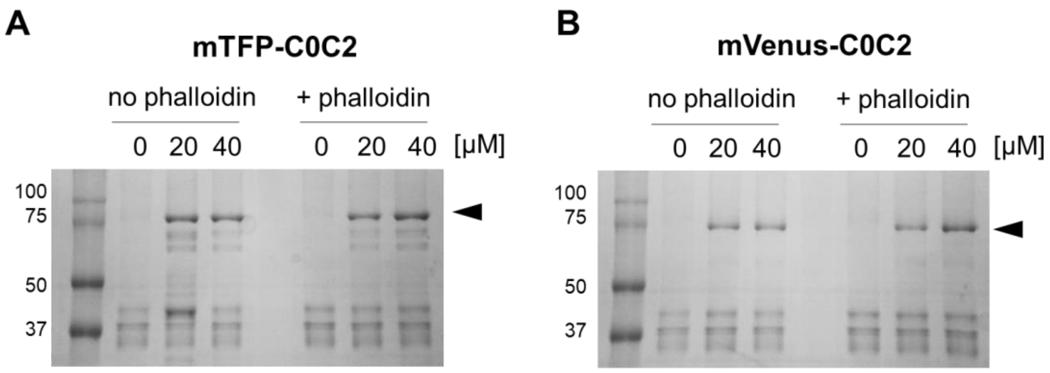

Figure S6. Effect of Phalloidin-iFluor514 on (A) mTFP-C0C2 and (B) mVenus-C0C2 binding to isolated native thin filaments.

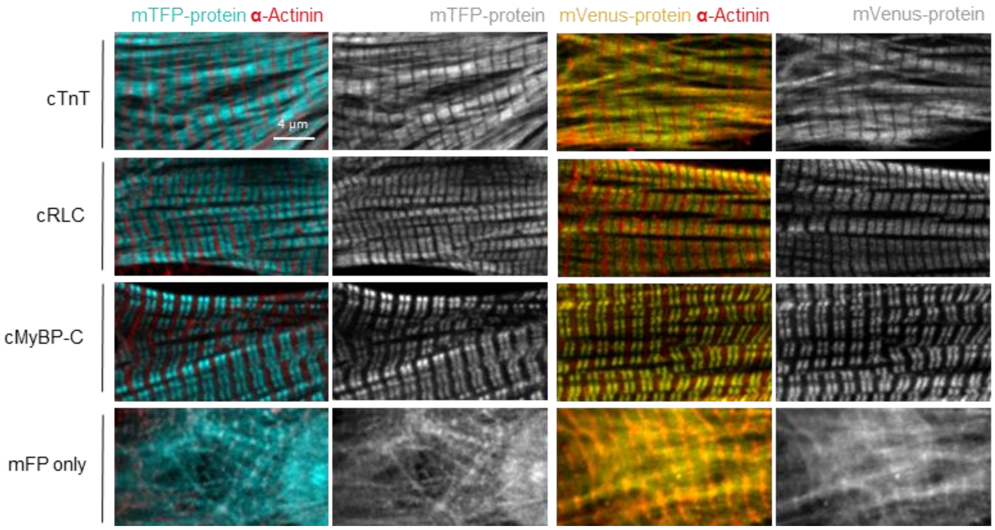

Fig. S7. Confocal images showing the localization of all mTFP- (cyan) and mVenus-fusion proteins (orange) used in this study. NRCs were counterstained for a-actinin (red) to visualize Z-disks.

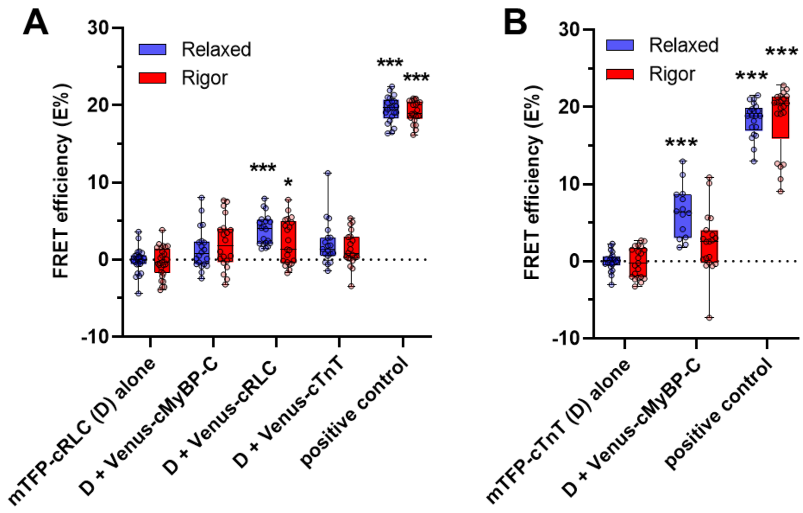

Fig. S8. Average FRET efficiencies for (A) mTFP-cRLC and (B) mTFP-cTnT in the presence of various acceptor molecules expressed in NRCs. Means ± SEM, n=14-20 cells from n=3-6 preparations. Statistical significance of differences vs donor alone in relaxing or rigor conditions were assessed with a two-way ANOVA followed by Sidak's post-hoc test: ***p<0.001.
